# Gastrointestinal injury in cardiopulmonary bypass: current insights and future directions

**DOI:** 10.3389/fphar.2025.1542995

**Published:** 2025-04-28

**Authors:** Qi-Long Mao, Zi-Hang Yu, Liang Nie, Fei-Xiang Wang, Yu-Hui Dong, Xiao-Fei Qi

**Affiliations:** ^1^ Department of Anesthesiology, Bazhong Central Hospital, Bazhong, Sichuan, China; ^2^ Department of Anesthesiology, Fushun County People’s Hospital, Zigong, Sichuan, China; ^3^ Department of Anesthesiology, The Affiliated Hospital, Southwest Medical University, Luzhou, Sichuan, China; ^4^ Department of Anesthesiology, Shenzhen Maternity and Child Healthcare Hospital, Southern Medical University, Shenzhen, Guangdong, China; ^5^ Department of Anesthesiology, Women and Children’s Medical Center, Shenzhen Maternity and Child Healthcare Hospital, Southern Medical University, Shenzhen, Guangdong, China

**Keywords:** gastrointestinal injury, cardiopulmonary bypass, multi-organ dysfunction, microbiota, metabolic products

## Abstract

Cardiopulmonary bypass (CPB) is an essential component of cardiac surgery. As CPB technology continues to advance and innovate, it has enabled the expansion of surgical boundaries and the resolution of many previously inoperable challenges. However, the occurrence of various complications during CPB warrants attention, with their prevention and management being paramount. The gastrointestinal tract, directly connected to the external environment, is vulnerable not only to external factors but also to internal changes that may induce damage. Both preclinical and clinical research have demonstrated the incidence of gastrointestinal injuries following CPB, often accompanied by dysbiosis and abnormal metabolic outputs. Currently, interventions addressing gastrointestinal injuries following CPB remain insufficient. Although recent years have not seen notable progress in this field, emerging academic research underscores the essential role of the gut microbiome and its metabolic products in sustaining overall health and internal equilibrium. Notably, their significance as the body’s “second genome” is increasingly recognized. Consequently, reevaluating the gastrointestinal damage post-CPB, alongside the associated dysbiosis and metabolic disturbances, is imperative. This reassessment carries substantial theoretical and practical implications for enhancing treatment strategies and bettering patient outcomes after CPB. This review aims to deliver a comprehensive synthesis of the latest preclinical and clinical research on CPB, address current challenges and gaps, and explore potential future research directions.

## 1 Introduction

Cardiopulmonary bypass (CPB) was first introduced in 1951, becoming a critical component in cardiac surgery, performing intricate biomechanical engineering tasks. The ongoing innovation and development of CPB technology have broadened the scope of medical procedures, enabling the resolution of previously insurmountable surgical challenges. However, as with any complex bioengineering system, CPB implementation encounters numerous challenges, including those related to safety and biomechanics, which demand substantial attention and resolution (Thomas and Beaudouin, 1951; Gerstein et al., 2022). CPB technology is tailored to meet diverse patient-specific needs and characteristics, encompassing various subcategories such as CPB for adults and for pregnant women. This level of customization not only showcases the precision and humanization of medical technology but also requires medical personnel to adapt the CPB protocols and procedures dynamically according to the unique pathophysiological conditions of each patient to ensure both safety and effectiveness of surgeries. Additionally, vigilant monitoring of patient conditions to timely identify and manage potential complications is crucial in enhancing patient survival rates and improving prognostic outcomes (Kapoor, 2014; Pouard and Bojan, 2013; Kunst et al., 2019).

The gastrointestinal tract, as an organ directly linked to the external environment, is susceptible to both external influences, such as trauma that can lead to gastrointestinal perforation, and internal changes, such as gut injury following acute brain damage (Revell et al., 2018; De Rosa et al., 2024). Notably, the intestinal microbiota and their metabolic products, termed the “second genome,” play a pivotal role as mediators in multi-organ damage. They can propagate damage from other injured organs and reciprocally impact the function of other organs through gastrointestinal disturbances (Huo and Wang, 2024; Zhou et al., 2024; Wen et al., 2020; Wang H. et al., 2013). Research indicates that gastrointestinal injuries post-CPB are predominantly caused by mucosal ischemia, leading to complications including mesenteric ischemia, pancreatitis, cholecystitis, and intestinal obstruction. Ischemia, induced by inadequate perfusion during CPB, significantly contributes to systemic inflammation, hypothermia, and mechanical stress. Additionally, non-ischemic mechanisms such as bacterial translocation, adverse drug reactions, and iatrogenic organ damage also play important roles (Allen, 2014). Given the crucial mediating role of gastrointestinal injuries in multi-organ damage post-CPB and the current inadequacy of interventions, there is a pressing need for further research to enhance these measures. This review aims to summarize the current understanding and future directions of post-CPB gastrointestinal injury from both basic and clinical perspectives, with special attention to differentiating the effects of various CPB modalities.

## 2 Post-CPB gastrointestinal injury: a crucial mediator in multi-organ dysfunction

### 2.1 Mechanisms of gastrointestinal injury in different CPB modalities

It is critical to recognize that different CPB techniques produce varying degrees and mechanisms of gastrointestinal injury. Standard CPB with aortic cross-clamping causes transient intestinal hypoperfusion and reperfusion effects, while deep hypothermic circulatory arrest (DHCA) introduces more severe ischemia-reperfusion injury due to complete circulatory cessation (Allen, 2014; Hessel, 2004). Partial bypass maintains some native perfusion but still alters intestinal microcirculation significantly (Sack et al., 2002a). CPB required for surgical repair of congenital heart disease induces a systemic inflammatory response worsening intestinal dysbiosis and leading to intestinal epithelial barrier dysfunction (Owens et al., 2024). Selective cerebral perfusion during aortic procedures reduces but does not eliminate intestinal injury (Algra et al., 2012). These distinctions are crucial for understanding the pathophysiology and developing targeted interventions.

Ischemia, the primary inducer of gastrointestinal complications, involves mechanisms including insufficient visceral perfusion and impaired oxygenation. Under normal physiological conditions, the visceral circulation receives approximately 20% of cardiac output and consumes a similar proportion of total oxygen, which is essential for normal abdominal organ function. This circulatory system utilizes key arterial branches such as the celiac, superior mesenteric, and inferior mesenteric arteries—all originating from the abdominal aorta—to establish an efficient blood supply network (Allen, 2014).

**FIGURE 1:**
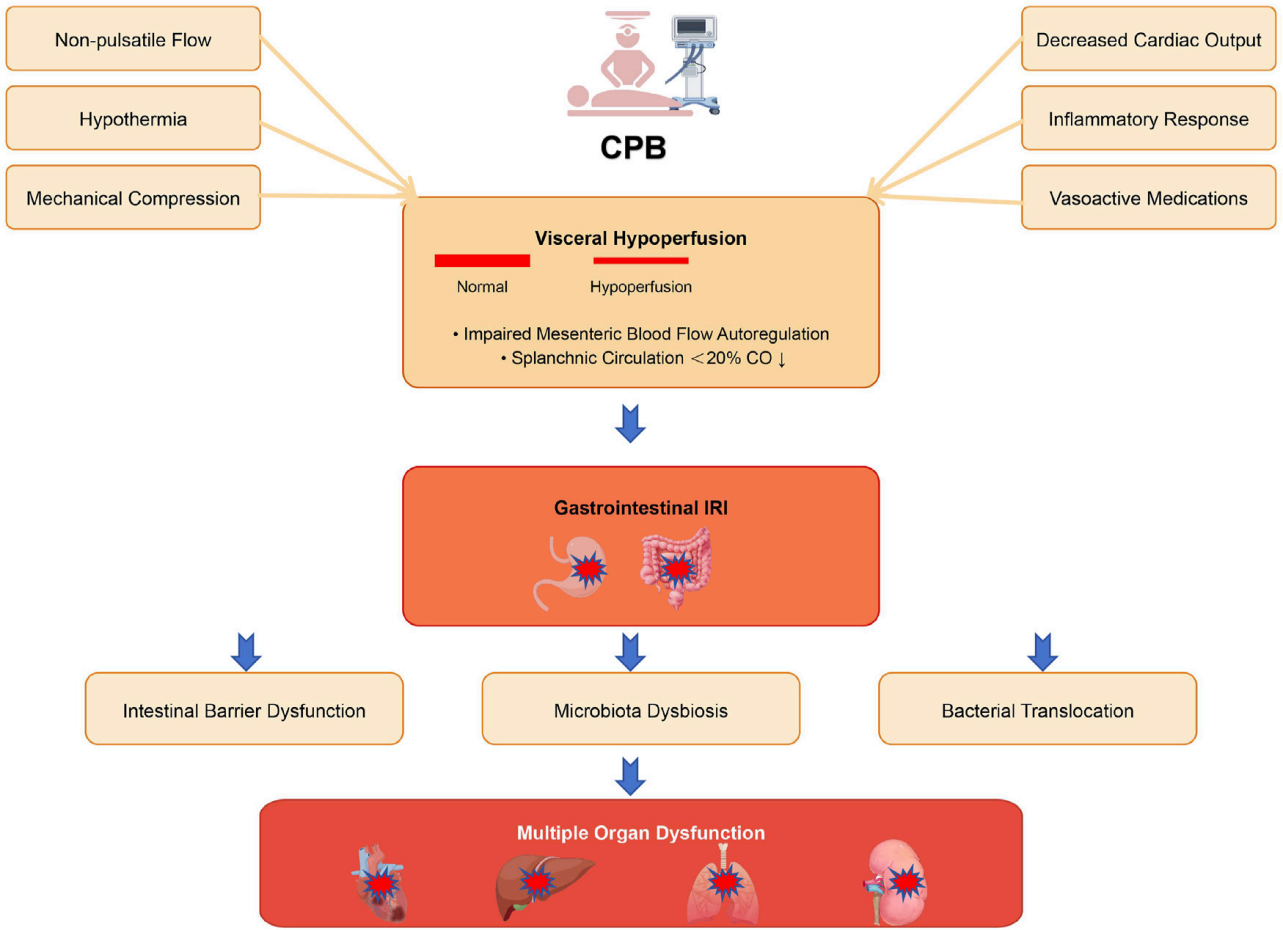
Schematic illustration of the mechanisms underlying gastrointestinal injury during CPB. CPB initiates multiple pathophysiological processes that compromise intestinal integrity. Non-pulsatile flow, hypothermia, mechanical compression, decreased cardiac output, inflammatory response, and vasoactive medications collectively lead to visceral hypoperfusion, characterized by impaired mesenteric blood flow autoregulation and reduced splanchnic circulation. This hypoperfusion subsequently triggers gastrointestinal IRI, resulting in intestinal barrier dysfunction, microbiota dysbiosis, and bacterial translocation. These pathological alterations ultimately contribute to multiple organ dysfunction, affecting the heart, lungs, liver, and kidneys.

The visceral circulation acts as a sophisticated “blood reservoir,” capable of rapidly initiating compensatory mechanisms in response to emergencies such as low blood volume, elevated catecholamine levels, or sharp declines in cardiac output, thereby stabilizing vital signs. However, this compensatory ability is not limitless. Under persistent high-stress conditions during cardiac surgery, CPB operations, or cardiogenic shock, the capacity of the visceral circulation to maintain adequate perfusion may be compromised, potentially leading to visceral ischemia (Hessel, 2004) (Figure 1).

**FIGURE 2:**
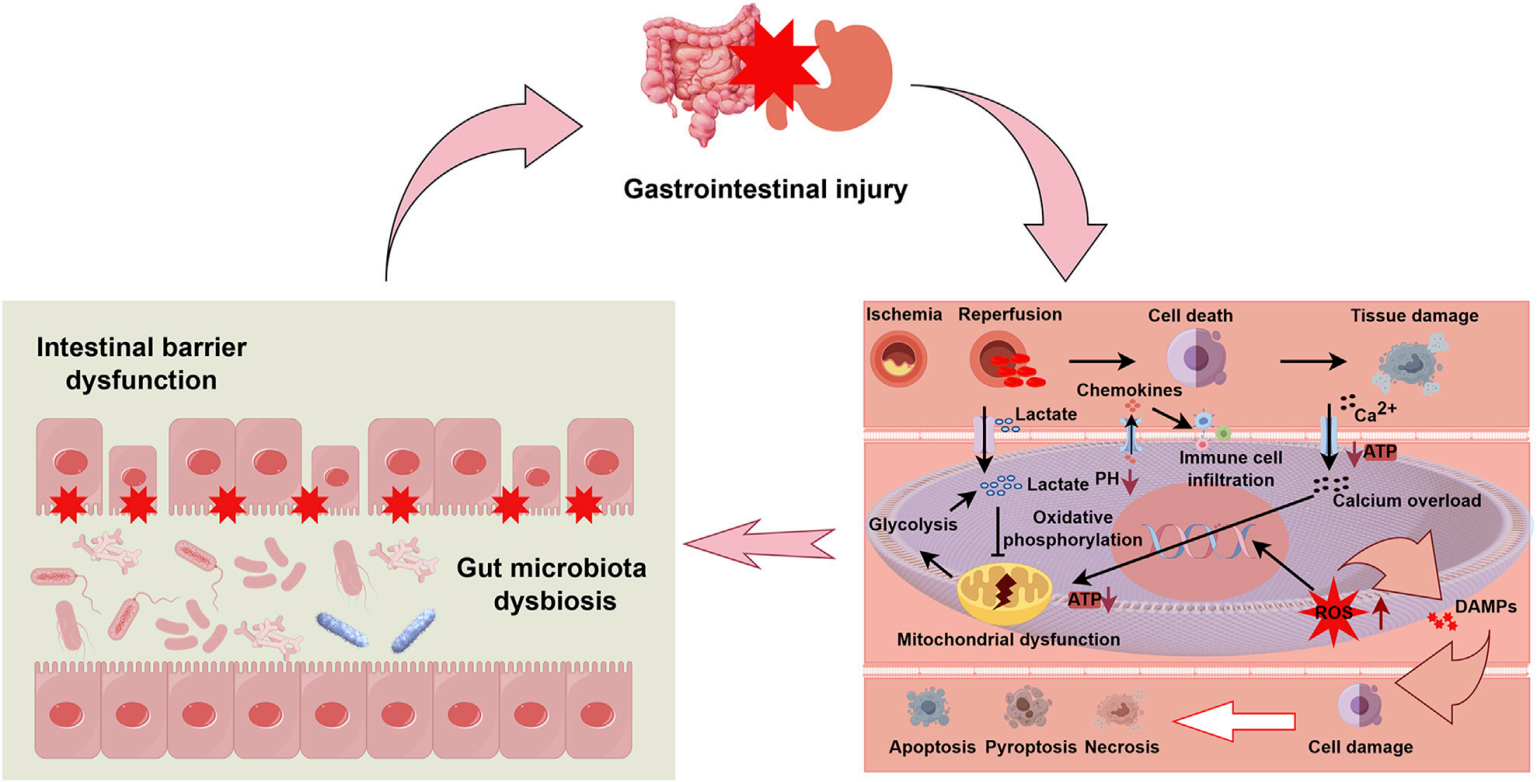
Integrated mechanisms of gastrointestinal injury following ischemia-reperfusion. This schematic diagram illustrates the pathophysiological cascade of gastrointestinal injury with emphasis on three interconnected components. The upper section depicts overall gastrointestinal injury as represented by affected intestinal and gastric organs. The left lower panel demonstrates two critical consequences of ischemia-reperfusion injury (IRI): intestinal barrier dysfunction, characterized by disruption of tight junctions between enterocytes (indicated by red stars), and gut microbiota dysbiosis, shown as altered microbial composition within the intestinal lumen. The right lower panel details the cellular and molecular mechanisms of IRI: initial ischemia triggers anaerobic glycolysis, lactate accumulation, and pH reduction. Upon reperfusion, paradoxical exacerbation of injury occurs through several pathways: (1) mitochondrial dysfunction with impaired ATP production and oxidative phosphorylation, (2) intracellular calcium (Ca^2+^) overload, (3) generation of reactive oxygen species (ROS), and (4) release of damage-associated molecular patterns (DAMPs). These events promote chemokine secretion, immune cell infiltration, and activation of multiple cell death pathways (apoptosis, pyroptosis, and necrosis), ultimately culminating in tissue damage. The bidirectional arrows between panels indicate the cyclic and self-reinforcing nature of these pathological processes, where barrier dysfunction and dysbiosis both contribute to and result from cellular injury mechanisms. Abbreviations: ATP, adenosine triphosphate; DAMPs, damage-associated molecular patterns; ROS, reactive oxygen species; IRI, ischemia-reperfusion injury.

The regulatory functions of blood supply in the mesentery are predominantly managed by resistance arterioles. These arterioles dynamically adjust their diameter in response to fluctuations in mean arterial pressure and metabolite accumulation, thus precisely regulating blood flow. Normally, mesenteric blood flow is auto-regulated and can be redistributed to prioritize critical areas such as the intestinal villi. However, under extreme stress or abrupt flow changes during CPB, this auto-regulation may fail, rendering the visceral circulation particularly vulnerable (Allen, 2014; Hessel, 2004). Notably, recent studies by Maeda et al. have demonstrated that ischemia-reperfusion injury triggers a complex cascade of cellular responses including activation of inflammatory mediators, increased oxidative stress, and programmed cell death, which collectively contribute to intestinal mucosal damage (Maeda and Ruel, 2015). Abboud et al. revealed that the intestinal villi undergoes significant structural and functional alterations during ischemia-reperfusion, contributing to impaired nutrient absorption and barrier dysfunction (Abboud et al., 2008). These results further enhance our understanding of the pathological mechanisms underlying gastrointestinal ischemia-reperfusion injury (IRI) at the basic research level. A simplified schematic representation of the mechanism of gastrointestinal IRI is presented in Figure 2.

Additional factors contributing to inadequate visceral perfusion include reductions in cardiac output, local flow obstructions, and insufficient systemic mean arterial pressure. Complicating elements like systemic inflammation, non-pulsatile blood flow, hypothermia, specific drug side effects, and mechanical compression can also exacerbate the risk of visceral ischemia (Dery et al., 2023; Sakorafas and Tsiotos, 1999; Dai et al., 2024). These interconnected factors collectively lead to gastrointestinal IRI, which not only damages the gastrointestinal tract itself but can also trigger multi-organ dysfunction.

### 2.2 Impact on intestinal microbiota and barrier function

During ischemic events, the reduction in blood and oxygen supply to the intestines rapidly affects tissue integrity. Subsequent reperfusion often exacerbates damage due to reactive oxygen species and inflammatory mediator accumulation, significantly compromising intestinal barrier integrity. This disruption triggers extensive changes in the intestinal microbiota composition. These changes may allow previously confined intestinal microbiota and their metabolic products to enter the bloodstream, initiating a series of reactions resulting in multi-organ damage (Chen et al., 2022).

Changes in the intestinal microbiota post-IRI are rapid and evident within just 1 h, with significant alterations observed in the colon microbiota, including increases in populations of *Escherichia coli* and *Prevotella*, as well as *Lactobacilli* (Wang et al., 2012). Similar changes are noted in the ileal microbiota during the early stages of intestinal IRI reperfusion, with clear differences manifesting after 12 h (Wang F. et al., 2013). Moreover, 16S rRNA and metabolomics studies have shown profound changes in the colonic bacterial community composition after intestinal IRI, with increased relative abundance of *Firmicutes* and *Bacteroidetes*, while *Verrucomicrobia* decreased. At the genus level, the abundance of *Bacteroidetes* and *Distasonis* increases. These microbial shifts influence metabolic activities within the microbiome, particularly affecting genes related to secondary metabolite biosynthesis and polysaccharide metabolism (Deng et al., 2022).

Recent data in pediatric patients demonstrates that microbiome dysbiosis can persist for up to 1 year following CPB procedures, highlighting the long-term consequences of these alterations (Yin et al., 2025). This prolonged dysbiosis may contribute to the 20%–40% rate of gastrointestinal complications observed in pediatric cardiac surgery patients, ranging from necrotizing enterocolitis to feeding intolerance and chronic malnutrition (Owens et al., 2024; Yin et al., 2025).

These findings illustrate that during CPB, the significant reduction in blood perfusion renders the patient’s gastrointestinal system particularly vulnerable to IRI. This IRI not only directly impairs gastrointestinal tissues but also induces significant alterations in the intestinal ecosystem. These shifts in the intestinal microbiota and their metabolic products exert profound physiological and pathological effects, influencing local gut function and potentially affecting distant organs through systemic circulation.

## 3 Current state of preclinical research on gastrointestinal damage post-CPB

### 3.1 Disturbances in intestinal microcirculation post-CPB

Despite their relative rarity, gastrointestinal complications following CPB are significantly linked to high mortality rates. During and post-CPB, disruptions in intestinal perfusion are frequently the harbingers of severe outcomes, such as multi-organ failure. Research involving long white pigs has shown that even with stable hemodynamic parameters during CPB, implementing partial normothermic left heart bypass markedly disturbs microvascular perfusion in the small intestine and significantly reduces blood flow velocity in post-capillary venules (Sack et al., 2002a). Additional studies on CPB animal models have revealed prevalent small arterial vasoconstriction, diminished blood flow velocity, and reduced functional capillary density, occurring concurrently with increased albumin leakage and leukocyte accumulation in the intestinal wall (Dong et al., 2009). Furthermore, research involving pigs has demonstrated that both extended minimally invasive extracorporeal circulation and conventional CPB induce intestinal mucosal damage, highlighting the vulnerability of the intestine to different CPB techniques (Rimpiläinen et al., 2011).

The effect of CPB on intestinal microvessel permeability is also noteworthy. With CPB initiation, there is a moderate increase in intestinal microvessel permeability, leading to heightened intestinal tissue water content. This increase is attributed not to a rise in capillary pressure but to alterations in vascular permeability itself (Cox et al., 1999). Studies have documented significant physiological changes in mucosal perfusion, epithelial permeability, edema formation, and blood flow regulation associated with CPB, necessitating comprehensive evaluation of their combined impact on intestinal function (Tofukuji et al., 2000).

Moreover, subtle adjustments in the spatial positioning of intestinal tissues critically influence intestinal damage mechanisms. Studies have indicated that even with adequate systemic blood supply, the use of roller pumps during CPB induces significant structural and functional changes in the muscle and mucosal layers of the small intestine (Kalder et al., 2015). While traditional thinking favored roller pumps, recent propensity-matched studies comparing patients on extracorporeal membrane oxygenation (ECMO) have suggested that centrifugal pumps may actually be associated with higher complication rates (Ündar et al., 2023), highlighting the complex relationships between pump design and end-organ perfusion.

### 3.2 Activation of immune cells and apoptosis in the progression of intestinal damage Post-CPB

Heart surgeries, particularly those involving CPB and DHCA, are closely linked to systemic inflammatory responses that significantly influence postoperative morbidity and mortality. Abnormal intestinal perfusion is often a direct consequence of these systemic inflammatory responses. The role of intestinal mast cells is pivotal in this context. Under cellular stress, these cells rapidly secrete various pre-formed effectors that significantly influence both local and systemic inflammatory responses. Research has shown that intestinal IRI is a primary pathophysiological event in rat models of DHCA, with mast cell activation playing a central role in both intestinal damage and resulting inflammatory responses (Karhausen et al., 2013).

Studies examining leukocyte depletion effects on the horse intestine after low-flow IRI induced by extracorporeal circuits suggest that while leukocyte depletion may reduce inflammatory responses in some cases, its impact on mitigating low-flow IRI effects on the horse’s small intestine is limited (Van Hoogmoed et al., 2001). Additional research investigating neutrophils’ role in mucosal perfusion disturbances post-CPB revealed that neutrophil isolation did not correlate strongly with these disturbances (Jormalainen et al., 2009).

In complex clinical settings involving ECMO and CPB, neonates and infants are particularly vulnerable to severe physiological challenges. Prolonged exposure to the CPB environment significantly heightens their risk of developing systemic inflammatory response syndrome and multi-organ dysfunction. Elevated circulating levels of intestinal fatty acid-binding proteins (FABPs) have emerged as sensitive indicators of intestinal epithelial damage. Further research has indicated that levels of cleaved caspase-8 increase significantly in the intestinal epithelial cells of damaged piglets, suggesting that epithelial cell apoptosis may be an early and crucial factor in the intestinal mucosal damage observed in ECMO models (MohanKumar et al., 2014). Future studies are essential to fully elucidate the role of apoptosis in post-CPB intestinal damage and to provide more scientifically sound and effective guidelines for clinical practice.

### 3.3 Improving treatment measures for intestinal damage Post-CPB

#### 3.3.1 Monitoring intestinal damage post-CPB

Timely and effective monitoring of intestinal damage after CPB is essential for optimizing patient outcomes. In this context, dopexamine, a weak-beta 2 agonist, initially garnered attention for its potential to enhance blood flow and support monitoring functions. However, studies across different animal models have shown variable results. In sheep models under hypothermic CPB conditions, dopexamine administration did not significantly improve intestinal conditions compared to placebo (Flynn et al., 2003; Madorin et al., 1999; Bangash et al., 2013; Stamler et al., 1998). Conversely, in pigs subjected to partial left heart bypass CPB, dopexamine significantly reduced intestinal perfusion injury (Sack et al., 2002b), and in rabbits, improved laser Doppler velocimetry readings of the jejunum and ileum (Bastien et al., 1999). These varied findings suggest that dopexamine’s impact on CPB-related intestinal injuries can be inconsistent across different preclinical CPB animal models.

Additionally, significant changes in microRNA (miRNA) expression have been observed in post-CPB intestinal injury studies, suggesting that miRNAs may serve as potential biomarkers for assessing intestinal damage. In a piglet model of DHCA, comparative analyses identified differential expression of 25 miRNAs between study and control groups, with changes in miR-122 being particularly prominent (Lin et al., 2015). The role of other miRNAs or non-coding RNAs in intestinal injury following extracorporeal circulation still requires further investigation. Future research should focus on translating these molecular markers into clinical practice to enhance the safety and outcomes of CPB procedures.

#### 3.3.2 Intestinal microbiota and their metabolic products

The interaction between intestinal microbiota and their metabolic products with intestinal IRI has been comprehensively validated through numerous studies (Wang et al., 2023; Deng et al., 2023; Deng et al., 2021a). This relationship is particularly critical in the context of post-CPB intestinal damage. Advanced research has demonstrated that these metabolic products, including succinate, milnacipran, capsiate, indole-3-lactic acid, and pravastatin, significantly influence the intestinal damage process by modulating key biological signaling pathways such as TRPV1, Gpx4, YAP, Nrf2, and IL-13 (Wang et al., 2023; Deng et al., 2023; Deng et al., 2021a; Zhang et al., 2023; Deng et al., 2021b).

Furthermore, intestinal microbiota play a direct role in managing post-CPB intestinal damage, beyond their metabolic functions. Research has identified a significant correlation between preoperative fecal abundance of *Lactobacillus murinus* and postoperative intestinal IRI severity. Experimental evidence suggests that administering *Lactobacillus murinus* significantly reduces intestinal IRI-induced damage and improves survival rates in mice. Additionally, *in vitro* studies demonstrate that *Lactobacillus murinus* activates the TLR2 signaling pathway, promoting anti-inflammatory cytokine IL-10 release from macrophages, thereby providing protection against intestinal damage (Hu et al., 2022).

Given the significant role of intestinal microbiota and their metabolic products as the body’s “second genome” (Zhu et al., 2010), it is crucial to further explore their changes and regulatory mechanisms during CPB to enhance outcomes of post-CPB intestinal damage and improve survival rates.

#### 3.3.3 ECMO

ECMO provides essential support for patients with severe cardiopulmonary failure by offering continuous extracorporeal respiratory and circulatory assistance (White and Fan, 2016). Recent research using a porcine post-CPB model has highlighted ECMO’s efficacy in mitigating intestinal mucosal barrier damage by reducing inflammation and cellular apoptosis (Liang et al., 2019). In studies of acute respiratory distress syndrome in porcine models post-trauma, ECMO showed potential protective effects on the intestine, though initial exacerbation of mucosal damage was observed (Ni et al., 2015). Additional research involving rabbits demonstrated ECMO’s benefits in managing prolonged hemorrhagic shock, enhancing tissue perfusion rapidly and decreasing the inflammatory response, thus mitigating long-term intestinal damage (Zhao et al., 2014).

Beyond cardiopulmonary resuscitation, ECMO enhances intestinal function during organ transplantation. Studies involving porcine models for intestinal transplantation have shown that ECMO support significantly improves intestinal absorption function compared to control groups, reducing caspase-3 expression in the intestinal mucosa and decreasing cell apoptosis (Guo et al., 2020).

Moreover, innovative research has explored the synergistic potential of ECMO with continuous renal replacement therapy (CRRT) to address complications such as intestinal villi detachment, edema, and alterations in tight junctions and epithelial cell connectivity within the intestinal mucosa (He et al., 2014). This combination therapy provides a deeper understanding of the mechanisms of intestinal damage associated with ECMO and suggests ways to further mitigate such damage post-CPB.

In summary, while ECMO is instrumental in reducing post-CPB intestinal damage, it is also associated with some level of induced intestinal injury. However, the damage from ECMO is generally less severe than that caused by CPB alone, underscoring its unique benefits in managing post-CPB intestinal injuries. The potential additive therapeutic effects of combining ECMO with CRRT warrant further investigation to confirm and refine these findings.

#### 3.3.4 Other medicinal or treatment approaches

In addition to the primary interventions previously described, various pharmacological and non-pharmacological treatments are being explored for managing post-CPB intestinal injury. These include κ-opioid receptor agonists, penehyclidine hydrochloride, albumin, N-acetylcysteine, activated α7nAChR, cold-inducible RNA-binding protein, selectin-sialyl Lewis, and poly-ADP-ribose polymerase. Experimental studies have confirmed that these agents substantially enhance intestinal barrier function, improve mesenteric endothelial dysfunction, moderate inflammatory responses, and normalize mesenteric vascular permeability (Zhang et al., 2020; Sun et al., 2011; Doguet et al., 2012; Chen et al., 2018; Xu et al., 2014; Li et al., 2019; Cox et al., 2000; Szabó et al., 2004).

Non-pharmacological interventions also show significant benefits. For instance, research on low-temperature circulatory arrest in pigs demonstrated that a lower core temperature of 20°C was more effective than 30°C in reducing intestinal wall edema and inflammatory responses (Khaladj et al., 2011). Steroid administration such as prednisolone preserved normal capillary density and prevented arterial constriction, underscoring steroids’ potential to protect microvascular function (Sack et al., 2001).

Gas therapies have also shown promise. A study involving sheep undergoing CPB found that inhaled nitric oxide significantly supported intestinal function and structural integrity. This intervention helped restore intestinal function and crucially maintained red blood cell deformability, essential for effective blood circulation (Kamenshchikov et al., 2024). Additionally, a rabbit study on hypoxia-hyperoxia preconditioning discovered that this method effectively balanced nitric oxide metabolites and curbed excessive endothelin-1 production, thereby alleviating inflammation and enhancing organ protection (Mandel et al., 2020). Another study on pigs examined perfusion techniques during thoracoabdominal aortic aneurysm surgery, revealing that selective visceral perfusion and distal aortic perfusion each offered distinct benefits, though they did not entirely prevent intestinal tissue damage; they did reduce its severity and improved outcomes (Kalder et al., 2012).

Optimizing perfusion solutions during CPB also shows potential for advancing intestinal mucosal preservation. Research on horses demonstrated that a modified organ perfusion solution could maintain the integrity of the intestinal mucosa for up to 12 h without blood and oxygen supply, targeting specific IRI mechanisms and suggesting avenues for enhancing prophylactic strategies against post-CPB intestinal damage (Polyak et al., 2008).

**FIGURE 3:**
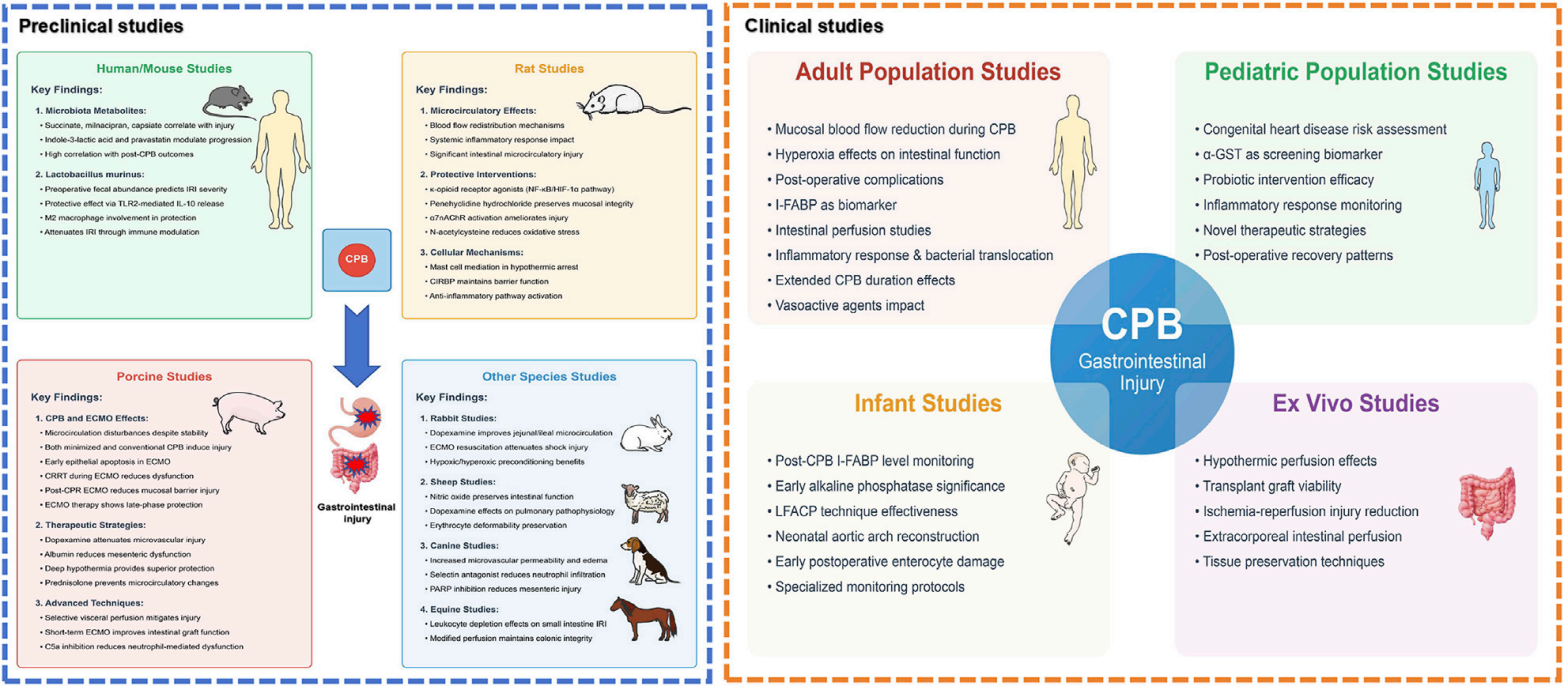
Comprehensive landscape of preclinical and clinical research on CPB-associated gastrointestinal injury. This schematic illustration presents a holistic view of both preclinical studies (left panel) and clinical research domains (right panel) investigating gastrointestinal injury following cardiopulmonary bypass (CPB). The preclinical studies encompass multiple animal models: Human/mouse studies demonstrate significant correlations between microbiota metabolites and post-operative outcomes, with *lactobacillus* murinus showing protective effects through TLR2-mediated pathways. Rat models reveal protective interventions including κ-opioid receptor agonists targeting NF-κB/HIF-1α pathways, penehyclidine hydrochloride, and α7nAChR activation, while highlighting the role of mast cells and CIRBP in intestinal barrier maintenance. Porcine studies, representing a substantial proportion of research, evaluate various CPB techniques and therapeutic strategies, particularly emphasizing microcirculatory alterations and benefits of interventions such as dopexamine and selective visceral perfusion. Additional species studies provide complementary insights: rabbit models validate preconditioning strategies and ECMO resuscitation; sheep studies assess nitric oxide and dopexamine effects; canine models investigate mechanisms of microvascular dysfunction; and equine studies examine leukocyte-mediated responses. The clinical research domains (right panel) illustrate four major areas surrounding the central focus of CPB-associated gastrointestinal injury: Adult population studies highlight investigations in mucosal blood flow alterations, hyperoxia effects, and post-operative complications; Pediatric population studies emphasize congenital heart disease risk assessment and biomarker screening using α-GST; Infant studies focus on specific monitoring protocols including I-FABP level monitoring and LFACP technique effectiveness; and *Ex vivo* studies showcase experimental approaches in tissue preservation and perfusion techniques. The interconnected nature of these research domains is represented through connecting pathways, emphasizing the translational continuum from preclinical models to clinical applications. Abbreviations: α7nAChR, alpha-7 nicotinic acetylcholine receptor; α-GST, alpha glutathione S-transferase; CIRBP, cold-inducible RNA-binding protein; CPB, cardiopulmonary bypass; CRRT, continuous renal replacement therapy; ECMO, extracorporeal membrane oxygenation; HIF-1α, hypoxia-inducible factor 1-alpha; I-FABP, intestinal fatty acid-binding protein; IL-10, interleukin-10; LFACP, low-flow antegrade cerebral perfusion; NF-κB, nuclear factor kappa B; PARP, poly-ADP-ribose polymerase; TLR2, Toll-like receptor 2.

In summary, preclinical CPB models have identified significant changes in intestinal mucosal integrity, vascular permeability, and barrier function post-CPB. The strategic application of treatments can improve diagnostic capabilities for early detection of intestinal damage. Intestinal microbiota and their metabolic byproducts play a critical role in treating post-CPB intestinal injuries. ECMO not only alleviates these injuries but also supports functional preservation of intestinal tissues. The current preclinical evidence regarding CPB-induced gastrointestinal injury is summarized in Table 1 and illustrated in Figure 3.

**TABLE 1:** Preclinical studies investigating gastrointestinal injury following CPB.

Interventions	Species	Key findings	References
Microbiota metabolites (succinate, milnacipran, capsiate, indole-3-lactic acid, pravastatin)	Human/Mouse	High correlation with post-CPB gastrointestinal injury; modulate injury progression	Zhou et al. (2024), Deng et al. (2023), Deng et al. (2021a), Zhang et al. (2023), Deng et al. (2021b)
*Lactobacillus* murinus	Human/Mouse	Preoperative fecal abundance correlates with post-operative intestinal IRI severity; attenuates IRI via TLR2-mediated IL-10 release from M2 macrophages	Hu et al. (2022)
CPB	Rat	Induces significant intestinal microcirculatory injury through blood flow redistribution and systemic inflammatory response	Dong et al. (2009)
Mast cells	Rat	Mediate intestinal injury and systemic inflammation during deep hypothermic circulatory arrest	Karhausen et al. (2013)
κ-opioid receptor agonists	Rat	Improve intestinal barrier function via NF-κB/HIF-1α pathway suppression	Zhang et al. (2020)
Penehyclidine hydrochloride	Rat	Preserves intestinal mucosal integrity post-CPB	Sun et al. (2011)
α7nAChR activation	Rat	Ameliorates CPB-induced intestinal injury	Chen et al. (2018)
N-acetylcysteine	Rat	Attenuates intestinal injury via oxidative stress and inflammatory response inhibition	Xu et al. (2014)
CIRBP	Rat	Maintains intestinal barrier function during deep hypothermic circulatory arrest	Li et al. (2019)
CPB variants	Porcine	Both minimized and conventional CPB induce mucosal injury	Rimpiläinen et al. (2011)
C5a inhibition	Porcine	Reduces neutrophil-mediated ileal microvascular dysfunction; no effect on mesenteric dysfunction	Tofukuji et al. (2000)
Roller pump ECC	Porcine	Induces significant intestinal muscular and mucosal alterations affecting perfusion	Kalder et al. (2015)
CPB-induced inflammation	Porcine	Low correlation between neutrophil activation and mucosal perfusion disturbances; distinct from classical IRI mechanisms	Jormalainen et al. (2009)
ECMO	Newborn porcine	Early epithelial apoptosis initiates gut mucosal injury	MohanKumar et al. (2014)
Dopexamine	Porcine	Attenuates CPB-induced intestinal microvascular injury	Sack et al. (2002b)
HCA	Porcine	Induces intestinal miRNA dysregulation and barrier dysfunction	Lin et al. (2015)
Post-CPR ECMO	Porcine	Reduces mucosal barrier injury post-ROSC	Liang et al. (2019)
ECMO therapy	Porcine	Exhibits late-phase protective effects on mucosal barrier	Ni et al. (2015)
Short-term ECMO	Porcine	Improves intestinal graft function from cardiac death donors	Guo et al. (2020)
CRRT during ECMO	Porcine	Reduces mucosal dysfunction and bacterial translocation	He et al. (2014)
Albumin supplementation	Porcine	Attenuates mesenteric vascular dysfunction and systemic inflammation	Doguet et al. (2012)
Deep hypothermia	Porcine	Superior intestinal protection compared to moderate hypothermia during HCA	Khaladj et al. (2011)
Prednisolone	Porcine	Prevents CPB-induced intestinal microcirculatory alterations	Sack et al. (2001)
Selective visceral perfusion	Porcine	Mitigates post-CPB intestinal injury	Kalder et al. (2012)
Dopexamine	Rabbit	Improves jejunal and ileal microcirculation during CPB	Bastien et al. (1999)
ECMO resuscitation	Rabbit	Attenuates hemorrhagic shock-induced intestinal injury via improved perfusion	Zhao et al. (2014)
Hypoxic/hyperoxic preconditioning	Rabbit	Ameliorates post-CPB intestinal injury	Mandel et al. (2020)
Dopexamine	Sheep	No improvement in intestinal function or post-CPB pulmonary pathophysiology	Stamler et al. (1998)
Nitric oxide	Sheep	Preserves intestinal function and erythrocyte deformability post-CPB	Kamenshchikov et al. (2024)
CPB initiation	Canine	Increases intestinal microvascular permeability and tissue edema	Cox et al. (1999)
Selectin antagonist TBC 1269	Canine	Reduces neutrophil infiltration without affecting microvascular permeability	Cox et al. (2000)
PARP inhibition	Canine	Reduces post-CPB mesenteric injury	Szabó et al. (2004)
Leukocyte depletion	Equine	No significant effect on low-flow IRI in small intestine	Van Hoogmoed et al. (2001)
Modified organ perfusion	Equine	Maintains colonic mucosal integrity for 12 h without blood/oxygen supply	Polyak et al. (2008)

Abbreviations: CPB, cardiopulmonary bypass; CIRBP, cold-inducible RNA-binding protein; CRRT, continuous renal replacement therapy; ECC, extracorporeal circulation; ECMO, extracorporeal membrane oxygenation; HCA, hypothermic circulatory arrest; IL-10, interleukin-10; IRI, ischemia-reperfusion injury; nAChR, nicotinic acetylcholine receptor; NF-κB, nuclear factor kappa B; PARP, poly-ADP-ribose polymerase; ROSC, return of spontaneous circulation; TLR2, Toll-like receptor 2.

## 4 Current clinical research on gastrointestinal injury post-CPB

### 4.1 Clinically relevant gastrointestinal injury post-CPB

In clinical settings, gastrointestinal injuries following CPB occur with notable frequency. A retrospective analysis spanning 8 years showed that out of 4,473 patients undergoing CPB surgery, 35 experienced gastrointestinal complications. These complications contributed to 22 deaths, representing 11.5% of the 191 total mortality cases during the study period (Huddy et al., 1991). Similar findings were reported by other studies, which observed CPB-related gastrointestinal injury rates ranging from 0.26% to 1% post-cardiac surgery, with mortality rates reaching up to 52% following such injuries (Schütz et al., 1998; Aouifi et al., 1999). CPB can significantly diminish mucosal blood flow and alter mesenteric perfusion due to primary endothelial dysfunction, with vasoconstrictors further reducing mesenteric perfusion (Ohri and Velissaris, 2006).

In pediatric populations, the incidence of gastrointestinal complications is significantly higher, ranging from 20%–40% of patients undergoing cardiac surgery with CPB. These complications span a spectrum from feeding intolerance to necrotizing enterocolitis and chronic malnutrition, substantially contributing to morbidity and increased hospital stay (Owens et al., 2024).

Additionally, substantial changes in intestinal permeability post-CPB have been observed, facilitating bacterial translocation. Studies have shown that post-CPB patients exhibit significantly altered intestinal mucosal permeability, demonstrated by an increased absorption ratio of lactulose to L-rhamnose compared to control groups (Sinclair et al., 1995; Ohri et al., 1993). After significant changes in intestinal permeability post-CPB, gut flora may enter the bloodstream, primarily indicated by elevated serum peptidoglycan levels (Tsunooka et al., 2004). Further research suggests a correlation between gastrointestinal mucosal injury, increased permeability, *Escherichia coli* bacteremia, and self-limiting inflammatory responses in elective coronary artery bypass grafting patients (Rossi et al., 2004).

Interestingly, another study noted that intestinal injuries occurred post-coronary artery surgery regardless of CPB involvement and could persist for approximately 5 days post-surgery (Ascione et al., 2006). Children undergoing CPB for congenital heart disease are also at risk for intestinal injuries, frequently presenting preoperative evidence of compromised intestinal epithelial integrity, with more severe conditions potentially mediating late postoperative epithelial barrier dysfunction (Typpo et al., 2015). These findings suggest that intraoperative hemodynamic changes are pivotal contributors to post-CPB intestinal injuries.

### 4.2 Evaluation metrics for gastrointestinal injury post-CPB

#### 4.2.1 Intestinal FABP (I-FABP)

FABPs are crucial in lipid transport systems, with specific subtypes expressed across various tissues. I-FABP, localized to the small intestine, is recognized for its sensitivity to intestinal damage and has been extensively studied as a potential clinical biomarker (Huang et al., 2022). Particularly in complex cardiac surgeries involving CPB, I-FABP serves as a significant marker. Studies have demonstrated that monitoring serum I-FABP levels during intensive care following CPB cardiac surgery provides an early, convenient, and objective predictor of patient prognosis (Zou et al., 2018). Additionally, research shows that I-FABP levels significantly increase during hypothermic circulatory arrest in aortic surgery patients undergoing CPB and quickly decrease upon reperfusion (Kano et al., 2017). Another study links intraoperative gastrointestinal injury with postoperative dysfunction and complications to 1-year mortality rates (Seilitz et al., 2021).

In the context of hemodialysis, correlations have been noted between I-FABP levels at intensive care unit admission and in-hospital mortality among CPB surgery patients undergoing hemodialysis, suggesting that inadequate intraoperative intestinal perfusion is a critical prognostic factor (Sekino et al., 2020). Elevated I-FABP levels in infants post-CPB indicate early epithelial cell injury, associated with the development of necrotizing enterocolitis within 6 h postoperatively (Watson et al., 2020).

In summary, I-FABP plays a pivotal role as a biomarker in monitoring intestinal injury following cardiac surgery, during hemodialysis, and in infants post-CPB. Changes in I-FABP levels reflect the severity and progression of intestinal damage, serving as an independent indicator of prognostic risk. Enhancing research and application of I-FABP is vital for advancing diagnostic and therapeutic standards and improving patient outcomes.

#### 4.2.2 Other evaluation metrics

Apart from I-FABP, other indices are crucial in evaluating intestinal injury post-cardiac surgery. A retrospective analysis indicated that intestinal ischemia often necessitates multiple abdominal surgeries and is a significant predictor of severity and 30-day mortality when two or more vasopressors are used (Wiesmueller et al., 2022). Additional findings relate plasma free hemoglobin levels and nitric oxide consumption during cardiac surgery to plasma I-FABP levels, suggesting that hemolysis-driven limitations in NO bioavailability are key factors in postoperative mucosal damage (Vermeulen Windsant et al., 2014). For patients with type IV thoracoabdominal aortic aneurysms, significant predictors of acute intestinal ischemia include renal insufficiency and visceral artery disease (Kieffer et al., 2008). Poor intestinal perfusion is also identified as a predictive factor for perioperative mortality in type A aortic dissection patients (Apaydin et al., 2002).

In pediatric populations, emerging indicators for predicting post-CPB intestinal injury include α-Glutathione S-transferase (αGST), which significantly rises in patients with prolonged bypass or aortic clamping times and clinical signs of intestinal injury (McMonagle et al., 2006). Additionally, research indicates a reduction in alkaline phosphatase (AP) activity during CPB in infants, with early low AP activity associated with postoperative support needs and organ dysfunction (Davidson et al., 2017).

Investigations into the relationship between intestinal injury, postoperative inflammatory response, and organ dysfunction reveal complexities. While some studies suggest that in low-risk cardiac surgeries, intestinal injury may not significantly affect inflammatory responses and organ dysfunction (Habes et al., 2023), others show a connection between prolonged CPB, elevated markers of intestinal injury, and inflammatory cytokines, though without a direct correlation to cytokine levels or gastrointestinal symptoms (Habes et al., 2017). Variations in surgical complexity, duration, and hemodynamic changes may significantly influence systemic cytokine responses and subsequent organ dysfunction.

These insights are invaluable, pointing to new dimensions in assessment and potentially enabling earlier detection and intervention in CPB-related gastrointestinal injuries, ultimately enhancing postoperative recovery and long-term patient outcomes.

### 4.3 Clinical interventions for gastrointestinal injury post-CPB

In clinical medicine, various interventions have been developed to mitigate gastrointestinal injuries in patients undergoing CPB. Research focusing on pediatric CPB patients indicates that probiotic supplementation significantly ameliorates dysbiosis by the seventh postoperative day, underscoring probiotics’ role in maintaining intestinal flora balance. However, no significant differences were observed in other intestinal health indicators such as fecal organic acid concentrations, bacterial translocation rates, and I-FABP levels between treatment and control groups (Toritsuka et al., 2024).

Non-pharmacological interventions have proven notably effective. For instance, low-flow antegrade cerebral perfusion during neonatal aortic arch reconstruction has been shown to decrease early renal and intestinal damage by optimizing blood flow (Algra et al., 2012). The use of centrifugal pump technology to maintain intestinal permeability post-CPB has been shown to be beneficial (Hyde et al., 1997), although recent evidence suggests more nuanced outcomes when comparing different pump types in ECMO patients (Ündar et al., 2023). The adoption of mini-CPB systems in myocardial revascularization has shown improved outcomes in terms of hemostatic system stability, reduced bleeding and transfusion requirements, decreased systemic inflammatory responses, and reduced renal and intestinal damage (Huybregts et al., 2007). Additionally, extracorporeal hypothermic perfusion devices equipped with intestinal perfusion modules have demonstrated effectiveness in intestinal transplantation (Muñoz-Abraham et al., 2016).

Collectively, these non-pharmacological interventions reveal a spectrum of possibilities for treating CPB-related gastrointestinal injuries. They enrich our understanding of the physiological and pathological changes occurring postoperatively, offer various effective treatment strategies, and underscore the necessity for future comprehensive, systematic research to discover timely interventions that could further improve the prognoses of CPB patients. A comprehensive overview of clinical investigations concerning CPB-associated gastrointestinal complications is presented in Table 2 and depicted in Figure 3.

**TABLE 2:** Clinical studies investigating gastrointestinal injury following CPB.

Study focus	Population	Key findings	References
Adult population studies			
Mucosal blood flow	Adults	CPB associated with severe reduction in intestinal mucosal blood flow	Dery et al. (2023)
Hyperoxia effects	Adults	Hyperoxia during critical illness adversely affects intestinal function	Dai et al. (2024)
CPB complications	Adults	Gastrointestinal complications occurred in 39 of 4,473 patients undergoing CPB surgery	Huddy et al. (1991)
Open heart surgery	Adults	Acute mesenteric ischemia occurs following open heart surgery	Schütz et al. (1998)
High-risk cardiac surgery	Adults	Post-CPB gastrointestinal complications predominantly occur in high-risk cardiac patients	Aouifi et al.(1999)
Multi-organ effects	Adults	Pulmonary and gastrointestinal injury detectable following uncomplicated CPB	Sinclair et al. (1995)
Intestinal function	Adults	CPB impairs small intestinal transport and increases intestinal permeability	Ohri et al. (1993)
Bacterial translocation	Adults	CPB-induced intestinal mucosal hypoperfusion leads to bacterial translocation	Tsunooka et al. (2004)
Inflammatory response	Adults	Correlation between mucosal injury, increased permeability, *E. coli* bacteremia, and inflammatory response activation without significant systemic circulation changes	Rossi et al. (2004)
Coronary surgery	Adults	Intestinal injury persists until postoperative day 5 in both on-pump and off-pump coronary surgery	Ascione et al. (2006)
I-FABP biomarker	Adults	I-FABP serves as predictor of acute gastrointestinal injury after cardiac surgery	Zou et al. (2018)
I-FABP monitoring	Adults	Plasma I-FABP monitoring valuable for detecting intestinal ischemia in cardiovascular surgery patients	Kano et al. (2017)
I-FABP correlation	Adults	I-FABP correlates with acute gastrointestinal injury post-cardiac surgery	Seilitz et al. (2021)
Hemodialysis patients	Adults	ICU admission enterocyte damage correlates with in-hospital mortality post-cardiac surgery in hemodialysis patients	Sekino et al. (2020)
Vasoactive agents	Adults	Administration of ≥2 vasoactive agents postoperatively strongly predicts intestinal ischemia severity and 30-day mortality	Wiesmueller et al. (2022)
Hemolysis effects	Adults	Cardiac surgery-associated hemolysis correlates with increased intravascular NO consumption and perioperative renal/intestinal injury	Vermeulen Windsant et al. (2014)
Type IV thoracoabdominal aneurysm	Adults	Renal dysfunction and visceral arterial disease predict acute intestinal ischemia	Kieffer et al. (2008)
Acute type A aortic dissection	Adults	Intestinal malperfusion identified as perioperative mortality risk factor	Apaydin et al. (2002)
Low-risk cardiac surgery	Adults	Intestinal injury not significantly involved in inflammatory response and organ dysfunction development after low-risk cardiac surgery	Habes et al. (2023)
Cytokine response	Adults	ECC duration contributes to cytokine response; intestinal injury not primary pathogenic factor	Habes et al. (2017)
Flow generation	Adults	Centrifugal pump technique improves post-CPB intestinal permeability	Hyde et al. (1997)
Mini CPB system	Adults	Miniaturized CPB reduces immediate post-operative intestinal tissue injury	Huybregts et al. (2007)
Kidney replacement therapy	Adults	Net ultrafiltration significantly correlates with gastrointestinal injury	Murugan et al. (2021)
Blood purification	Adults	Continuous blood purification reduces intestinal barrier dysfunction in MODS patients	Zhang et al. (2010)
Pediatric population studies			
Congenital heart disease	Children	Risk of intestinal injury in children undergoing CPB for congenital heart disease correction	Typpo et al. (2015)
α-GST biomarker	Children	α-GST potentially useful for screening post-cardiac surgery intestinal ischemic injury	McMonagle et al. (2006)
Probiotic intervention	Children	Probiotics mitigate CPB-induced intestinal injury in pediatric patients	Toritsuka et al. (2024)
Infant studies			
I-FABP levels	Infants	Elevated post-CPB I-FABP levels indicate early postoperative enterocyte damage	Watson et al. (2020)
Alkaline phosphatase	Infants	Early low alkaline phosphatase independently associated with subsequent postoperative support and intestinal dysfunction	Davidson et al. (2017)
*Ex vivo* studies			
Hypothermic perfusion	Human intestines	Extracorporeal intestinal perfusion maintains intestinal viability and reduces IRI in transplant grafts	Muñoz-Abraham et al. (2016)

Abbreviations: α-GST, Alpha glutathione S-transferase; CABG, coronary artery bypass graft; CPB, cardiopulmonary bypass; ECC, extracorporeal circulation; I-FABP, intestinal fatty acid-binding protein; ICU, intensive care unit; IRI, ischemia-reperfusion injury; LFACP, low-flow antegrade cerebral perfusion; MODS, multiple organ dysfunction syndrome; NO, nitric oxide.

## 5 Conclusions and perspectives

In clinical practice, gastrointestinal injuries are commonly observed both post-CPB and among critically ill patients. Research has highlighted that gastrointestinal injuries in patients undergoing CRRT should not be overlooked (Murugan et al., 2021). Studies have shown that compromised intestinal barrier function in patients with multiple organ dysfunction syndrome strongly correlates with adverse disease outcomes. CRRT not only improves clinical conditions but also enhances intestinal barrier function by inhibiting the degradation of occludin and ZO-1 (Zhang et al., 2010). While plasma measurements of tight junction proteins such as occludin and ZO-1 are utilized in some studies, it’s important to acknowledge the limitation that these markers are not intestine-specific, as they are also expressed in vascular endothelium, making it challenging to determine the intestinal contribution to their plasma levels (Zhang et al., 2010; Zeng et al., 2018). Research indicates that high oxygen inhalation in critically ill patients undergoing peripheral venous arterial ECMO treatment may trigger intestinal injury (Dai et al., 2024). Additionally, injuries to other organs can precipitate secondary intestinal damage (Nastos et al., 2016). These studies indicate that the etiology of gastrointestinal injury is multifaceted, necessitating a holistic research approach.

Current medical research, encompassing both preclinical experiments and clinical observations, consistently reports the occurrence of gastrointestinal injuries post-CPB. Although this area of research has diversified, significant advancements have been sporadic in recent years. In clinical settings, the symptoms of post-CPB gastrointestinal injury may be overshadowed by more severe symptoms, thus receiving inadequate attention. Nevertheless, the existence of post-CPB gastrointestinal injuries, which can lead to dysbiosis and metabolic disturbances, is undeniable. Recent scholarly focus has emphasized the crucial role of the gut microbiome and its metabolic products in maintaining health and homeostasis, notably as the body’s “second genome” (Zhu et al., 2010). Reevaluating the dysbiosis and metabolic disturbances resulting from post-CPB gastrointestinal injuries is therefore vital for refining treatment protocols and enhancing patient outcomes. The high operability and specificity of interventions targeting the gut microbiome make such measures especially pivotal for improving post-CPB gastrointestinal injuries.

With ongoing technological advances, particularly in microbiome testing and imaging techniques, new avenues have opened for thorough disease diagnosis and mechanism exploration. Some interventions, such as hypothermia therapy, perfusion techniques, and device enhancements, have proven effective in alleviating post-CPB gastrointestinal injuries. However, a significant gap remains in their clinical translation. Most existing clinical studies are observational, lacking large-scale, multicenter randomized controlled trials to definitively identify effective strategies for ameliorating post-CPB gastrointestinal injuries, particularly concerning the dynamics of the gut microbiome. The existing evidence confirming post-CPB gastrointestinal injuries, which may be subtle and asymptomatic in some clinical scenarios, underscores the significance of addressing these injuries and associated dysbiosis to improve CPB patient prognosis. Promoting the translation of preclinical research findings into clinical practice is expected to enhance outcomes for many CPB patients.

## Abbreviations

αGST, Alpha-Glutathione S-transferase; AP, Alkaline phosphatase; CPB, Cardiopulmonary bypass; CRRT, Continuous renal replacement therapy; DHCA, Deep hypothermic circulatory arrest; ECMO, Extracorporeal membrane oxygenation; FABPs, Fatty acid-binding proteins; I-FABP, Intestinal fatty acid-binding protein; IRI, Ischemia-reperfusion injury; miRNAs, MicroRNAs; NO, Nitric oxide.
